# Squalene in Nanoparticles Improves Antiproliferative Effect on Human Colon Carcinoma Cells Through Apoptosis by Disturbances in Redox Balance

**DOI:** 10.3390/ijms252313048

**Published:** 2024-12-04

**Authors:** Seyed Hesamoddin Bidooki, Javier Quero, Javier Sánchez-Marco, Tania Herrero-Continente, Inés Marmol, Roberto Lasheras, Victor Sebastian, Manuel Arruebo, Jesús Osada, María Jesús Rodriguez-Yoldi

**Affiliations:** 1 Departamento de Bioquímica y Biología Molecular y Celular, Facultad de Veterinaria, Instituto de Investigación Sanitaria de Aragón, Universidad de Zaragoza, E-50013 Zaragoza, Spain; h.bidooki94@gmail.com (S.H.B.); javiersanchezmarc@gmail.com (J.S.-M.); taniaherrero1992@gmail.com (T.H.-C.); josada@unizar.es (J.O.); 2 Departamento de Farmacología, Fisiología, Medicina Legal y Forense, Facultad de Veterinaria, Instituto de Investigación Sanitaria de Aragón, Universidad de Zaragoza, E-50013 Zaragoza, Spain; javierquero94@gmail.com (J.Q.); ines.marmol@gmail.com (I.M.); 3 Laboratorio Agroambiental, Servicio de Seguridad Agroalimentaria de la Dirección General de Alimentación y Fomento Agroalimentario, Gobierno de Aragón, E-50071 Zaragoza, Spain; rjlasheras@aragon.es; 4 Departamento de Ingeniería Química y Tecnologías del Medio Ambiente, Universidad de Zaragoza, E-50018 Zaragoza, Spain; victorse@unizar.es (V.S.); arruebom@unizar.es (M.A.); 5 Instituto de Nanociencia y Materiales de Aragón (INMA), CSIC, Universidad de Zaragoza, E-50009 Zaragoza, Spain; 6 Centro de Investigación Biomédica en Red de Bioingeniería, Biomateriales y Nanomedicina (CIBER-BBN), Instituto de Salud Carlos III, E-28029 Madrid, Spain; 7 Instituto Agroalimentario de Aragón, CITA, Universidad de Zaragoza, E-50013 Zaragoza, Spain; 8 CIBER de Fisiopatología de la Obesidad y Nutrición, Instituto de Salud Carlos III, E-28029 Madrid, Spain

**Keywords:** apoptosis, Caco-2 cells, nanoparticles, PLGA, ROS, squalene

## Abstract

Squalene, a triterpene found in extra virgin olive oil, has therapeutic properties in diseases related to oxidative stress, such as cancer. However, its hydrophobic nature and susceptibility to oxidation limit its bioavailability outside of olive oil. To expand its applications, alternative delivery methods are necessary. The objective of the present study was to examine the impact of squalene encapsulated in PLGA (poly(lactic-co-glycolic) acid) nanoparticles (PLGA + Sq) on the proliferation of human colon carcinoma Caco-2 cells, as well as its underlying mechanism of action. The findings demonstrated that PLGA + Sq exert no influence on differentiated cells; however, it is capable of reducing the proliferation of undifferentiated Caco-2 cells through apoptosis and cell cycle arrest in the G1 phase. This effect was initiated by the release of cytochrome c into the cytoplasm and the subsequent activation of caspase-3. Furthermore, squalene exhibited pro-oxidant activity, as evidenced by an increase in intracellular ROS (reactive oxygen species) levels. The results of the squalene effect on genes associated with cell death, inflammation, and the cell cycle indicate that its antiproliferative effect may be post-transcriptional. In conclusion, PLGA + Sq demonstrate an antiproliferative effect on Caco-2 cells through apoptosis by altering redox balance, suggesting squalene’s potential as a functional food ingredient for colorectal cancer prevention.

## 1. Introduction

Colon cancer represents the third most prevalent form of cancer and is the second leading cause of cancer-related mortality on a global scale [1,2]. The incidence of this cancer is significantly higher in developed countries, including those in Europe and the United States, in comparison to countries in Africa and Asia [3]. Patients with advanced colon cancer and metastasis continue to experience unfavorable prognoses, and drug resistance significantly impairs the therapeutic efficacy of chemotherapy. This is due to the resistance of colon carcinoma cells to chemotherapeutic drugs [1,4,5,6].

An altered redox status, accompanied by an elevated generation of reactive oxygen species (ROS), has been implicated in colon cancer [7,8,9,10]. Different studies revealed that fruits rich in phytochemicals with nutraceutical properties can affect the cell viability of colon cancer cell lines (HT29, Caco-2, and HCT116), inducing an imbalance in the cellular redox responses [11,12,13,14]. The alteration of this balance can act in two opposite ways in neoplastic cells: either fostering the cancer phenotype by enabling specific hallmarks, such as uncontrolled cell proliferation, migration, and survival; or triggering cell death by mechanisms such as apoptosis, autophagy, necrosis, or ferroptosis [2,7,10,13,15]. In light of these considerations, the utilization of natural compounds as alternative therapeutic modalities in the management of colon cancer has witnessed a notable surge [1,3,14].

The results of recent studies indicate that plants and other natural nutritional supplements may possess anticancer properties [3]. The Mediterranean dietary pattern has been proposed as a means of preventing disease due to its provision of a diverse range of plant-based food sources, including fruits, vegetables, olive oil, and nuts [16]. Virgin olive oil, the primary source of fat in this diet, contains a substantial amount of squalene, which acts as a natural antioxidant, anti-inflammatory, and antineoplastic agent against skin, colon, and lung cancer cells, as well as sarcoma [16,17]. Higher olive oil intake, particularly due to squalene, is linked to a reduced colorectal cancer risk through antioxidant and anticarcinogenic effects [18,19,20]. Research shows squalenoylated nanoparticles boost cisplatin’s effectiveness in colon cancer cells by enhancing drug uptake, cytotoxicity, ROS production, and apoptosis activation [21]. Squalene plays a role in the biosynthesis of phytosterols and terpenes in plants and cholesterol in animals [22]. It is absorbed by the body and accumulates in the liver as an isoprenoid lipid belonging to the terpene family [23]. It has a linear structure and six double bonds, which confer the ability to resist high temperatures during frying [17,22,24]. However, squalene, due to its hydrophobic nature, shows reduced bioavailability outside of olive oil and also oxidizes easily. Hence, in order to be able to use this compound outside of this aforementioned oil, new ways of administration are required [16].

The potential of nanotechnology to enhance existing methodologies while simultaneously diminishing the toxicity and unfavorable consequences associated with conventional treatments is considerable [25,26]. Consequently, a variety of naturally derived and biodegradable particles, including chitosan, poly(lactic-co-glycolic) acid (PLGA), and protein-based particles, have been developed [16,27,28]. It is acknowledged that emulsions and submicron emulsions have the potential to be effective alternatives for the delivery of squalene, due to their capability to enhance the bioavailability and stability of hydrophobic compounds. However, PLGA-based nanoparticles were selected for this study due to their distinctive capabilities in sustained and targeted delivery [29]. PLGA nanoparticles have been extensively documented for their biocompatibility and controlled release properties, which have the potential to prolong the bioactivity of squalene in a cellular environment, thereby overcoming its rapid oxidation. Furthermore, nanoscale encapsulation may facilitate enhanced cellular uptake, thereby offering a more precise delivery mechanism than emulsions [29,30]. In this study, to increase its action, squalene was loaded into PLGA nanoparticles. Materials based on PLGA are compounds that biodegrade within the body without causing toxicity as they are composed of two endogenous biological substances such as lactic acid and glycolic acid [16,31,32]. Overall, these particles loaded with squalene stabilized this compound and were employed to ascertain the impact of the said compound on several different metabolic and oxidative stress-related pathways in human colon cancer cells (Caco-2).

## 2. Results

### 2.1. The Vehicleization of Squalene and Cell Viability

**Figure 1:**
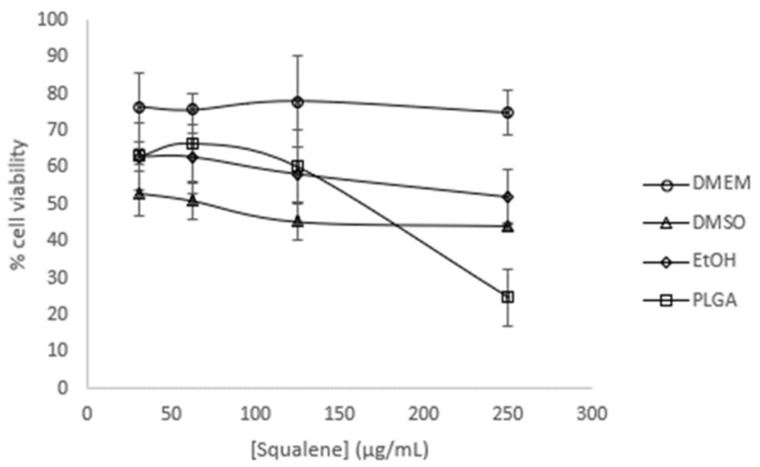
Viability effect on undifferentiated Caco-2 cells incubated with different squalene concentrations using DMEM, DMSO, EtOH, and PLGA vehicles for 72 h.

The initial objective was to study the potential impact of squalene on the viability of Caco-2 cells. Dose-response curves were constructed using varying concentrations of squalene (31.25, 62.5, 125, and 250 µg/mL) at a 72 h preincubation period, with different carriers such as DMEM (Dulbecco’s modified Eagle’s medium) as a control, 0.5% DMSO, 0.2% EtOH (75%), and PLGA-NPs (drug loading content: ≈0.6 squalene/PLGA (*w*/*w*)). The results demonstrated a slight dose-dependent antiproliferative effect of squalene (with the exception of DMEM), whereby PLGA-bound squalene exhibited the most notable reduction in cell viability (Figure 1).

**Figure 2:**
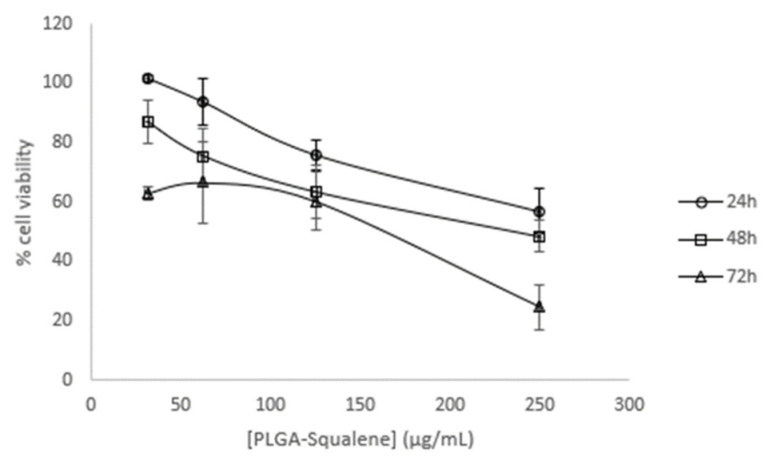
Time and dose-response viability effect of a range of PLGA + Sq on undifferentiated Caco-2 cells.

The PLGA vehicle was selected for further investigation, and dose-response curves were performed at the designated time points: 24, 48, and 72 h (Figure 2).

**Figure 3:**
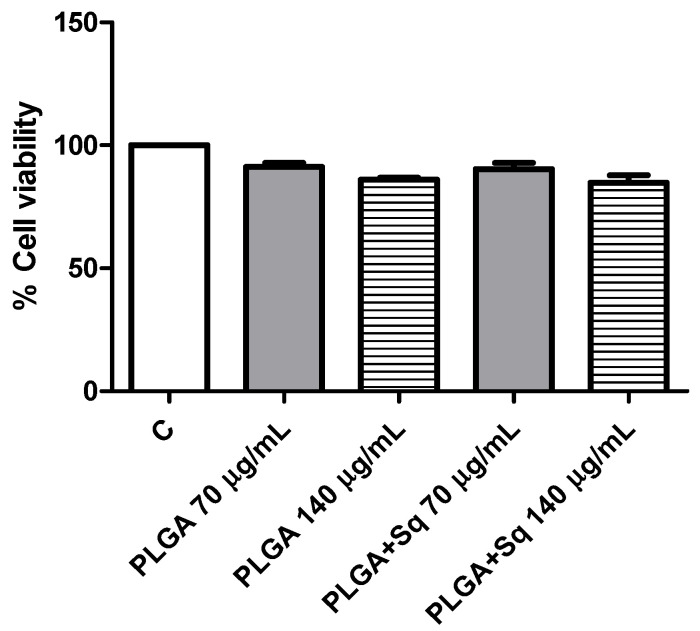
Differentiated Caco-2 cells incubated for 72 h with 70 or 140 μg/mL PLGA + Sq. PLGA 70 or 140 μg/mL (without Sq) represent the PLGA-NPs required for the indicated Sq concentration. C, control, refers to untreated cells.

The dose-response curves as functions of time demonstrated that the effect was more pronounced at extended times (72 h) and elevated concentrations (250 µg/mL). From the curve (Figure 2), the IC_50_ was calculated to be approximately 140 µg/mL, which was selected for subsequent tests. Similarly, to eliminate the potential for PLGA and squalene to exert adverse effects on non-cancerous cells, their viability was assessed in the presence of the vehicle with and without squalene at the aforementioned concentration. The results demonstrated that the differentiated cells were not significantly affected (Figure 3).

### 2.2. PLGA-Squalene Uptake in Caco-2 Cells

**Figure 4:**
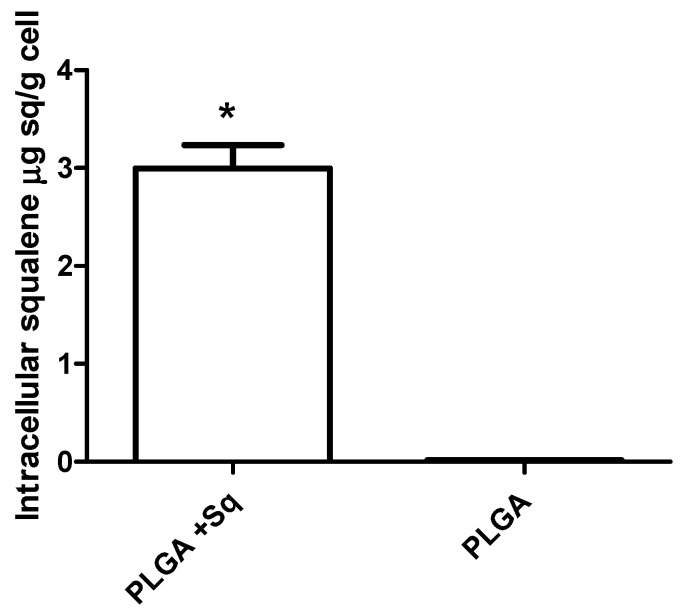
In vitro cellular uptake of squalene. Caco-2 cells were incubated with 140 μg/mL of PLGA + Sq NPs and PLGA NPs for a period of 24 h. * *p* < 0.05 vs. PLGA.

A cell uptake study is an indispensable methodology for evaluating the delivery potential of the nanoparticulated system. The cellular uptake was investigated through the examination of squalene extracted from Caco-2 cells via gas chromatography/mass spectrometry (GC/MS). To this end, the cells were treated with the previously obtained IC_50_ for PLGA-based squalene NPs and empty PLGA NPs after a brief incubation period. As illustrated in Figure 4, squalene entered the cells at 24 h, as evidenced by the decline in viability (Figure 2). These findings indicate that PLGA is an effective vehicle for delivering squalene in this cell line.

### 2.3. PLGA-Squalene on Cell Death Studies

Following the identification of squalene’s ability to enter cells, the subsequent objective was to ascertain the specific antiproliferative effect or mode of cell death exerted by this triterpene. Apoptosis represents a type of programmed cell death that can be induced by a wide variety of signals in cells. The intrinsic pathway, which is often activated by stressors such as DNA damage or oxidative stress, involves mitochondrial dysfunction and the release of cytochrome c into the cytosol [33]. The extrinsic pathway is initiated by death receptors on the cell surface, such as TNF receptors, which trigger the formation of a death-inducing signaling complex (DISC) and the subsequent activation of different caspases [34,35,36]. These signals ultimately result in the activation of executioner caspases, membrane vesicle formation, DNA fragmentation, and ultimately, cell death [33].

**Figure 5:**
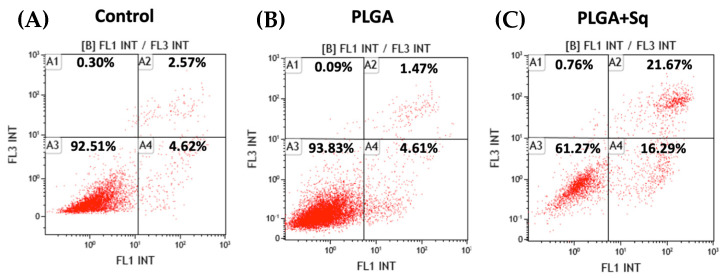
Incubation of undifferentiated Caco-2 cells for 72 h. (**A**) Negative control, referring to the untreated cells, (**B**) PLGA, (**C**) PLGA + Sq at IC_50_ concentration (140 μg/mL). Percentages of alive (A3), necrotic (A1), early apoptotic (A4) and late apoptotic (A2) cells are indicated.

Apoptosis studies were conducted via flow cytometry on cells treated with PLGA, with and without squalene, at a concentration corresponding to the IC_50_ at 72 h. The results demonstrated squalene-induced programmed cell death through both early and late apoptosis, thereby excluding the possibility of necrosis-mediated cell death, which could otherwise result in adverse effects such as inflammation (Figure 5). Furthermore, PLGA without squalene did not exert any effect on the cells, thereby excluding the aforementioned effect.

**Figure 6:**
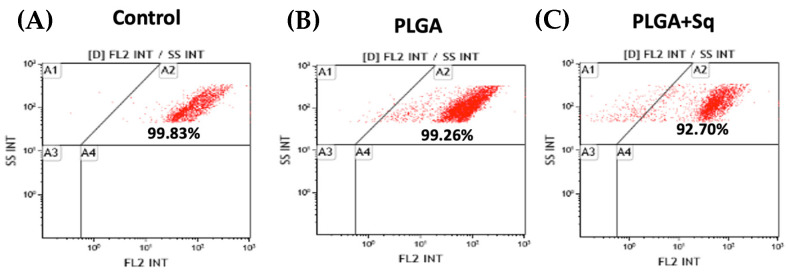
Undifferentiated Caco-2 cells with mitochondrial cytochrome c after 72 h incubation with/without PLGA or Sq (140 μg/mL). (**A**) Negative control, referring to the untreated cells, (**B**) PLGA, (**C**) PLGA + Sq. A1: cytochrome c released, A2: cytochrome c retained, A3 and A4: debris and dead cells.

**Figure 7:**
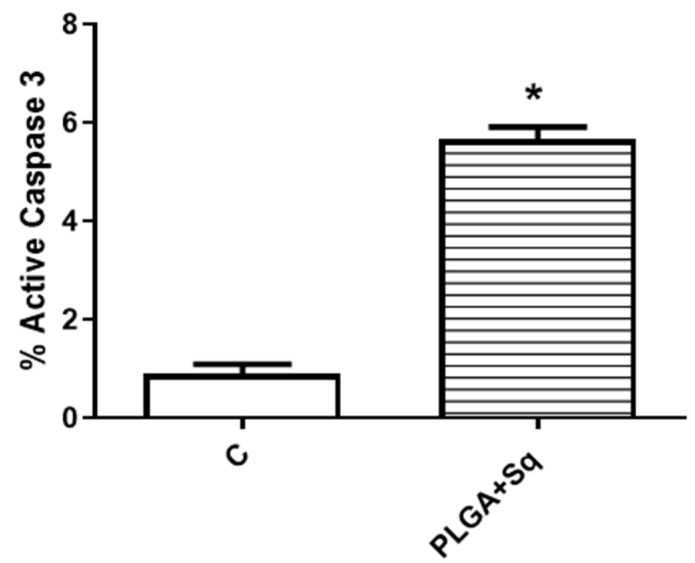
Percentage of undifferentiated Caco-2 cells with active caspase-3 after 72 h incubation with/without PLGA + Sq (140 μg/mL). * *p* < 0.05 vs. control.

Cytochrome c, present in the cytoplasm, plays a role in the activation cascade of caspases, including caspase-3, which is responsible for the execution of apoptosis [33]. Figure 6 illustrates that in cells treated with PLGA + Sq, the cytochrome c value is lower in the mitochondria. This indicates that the protein has been released from the mitochondria and may be involved in the process of apoptosis, as evidenced by the observations in Figure 5. Moreover, the activity of the apoptosis-executing caspase-3 was markedly elevated (Figure 7).

### 2.4. The Effect of PLGA-Squalene on Cell Cycle

**Figure 8:**
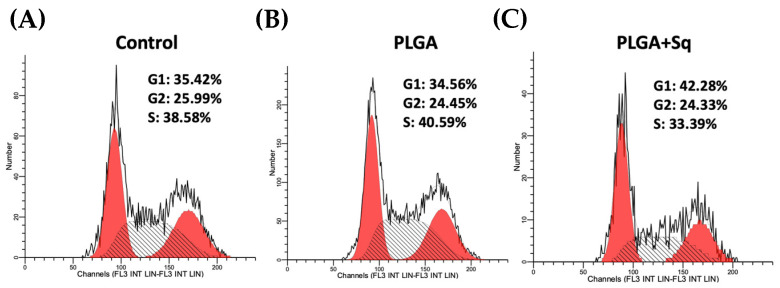
Measurement of the cell cycle after a 72 h incubation on undifferentiated Caco-2 cells (**A**) Negative control, referring to the untreated cells, (**B**) PLGA, (**C**) PLGA + Sq (IC_50_). From left to right: red peak: G1, black hatched peak: S, red peak: G2.

To study the impact of squalene on Caco-2 cells, RT-qPCR of the genes involved in cell death, inflammation, and the cell cycle (*NLRP3*, *PYCARD*, *BCAR1*, *IL-1B*, *GPX4*, *FAF1*, *RIPK1*, *MLKL*, *ATM*, *CHEK2*, *BECN1*, and *CCND1*) was developed. The results of the RNA expression analysis did not reveal any changes in the expression levels of the genes tested, which suggests that the observed antiproliferative effect of squalene may be post-transcriptional, rather than acting at the mRNA levels (Table S1, Figure S1). Accordingly, the potential impact of squalene on the cell cycle was investigated by examining the cell cycle under the aforementioned conditions (control, squalene, and PLGA + Sq at the IC_50_ concentration [140 µg/mL] for an incubation period of 72 h). The results demonstrated a slight cessation in the G1 phase, accompanied by a reduction in the S phase for the PLGA + Sq condition (Figure 8).

### 2.5. The Effect of PLGA-Squalene on ROS Intracellular Levels

**Figure 9:**
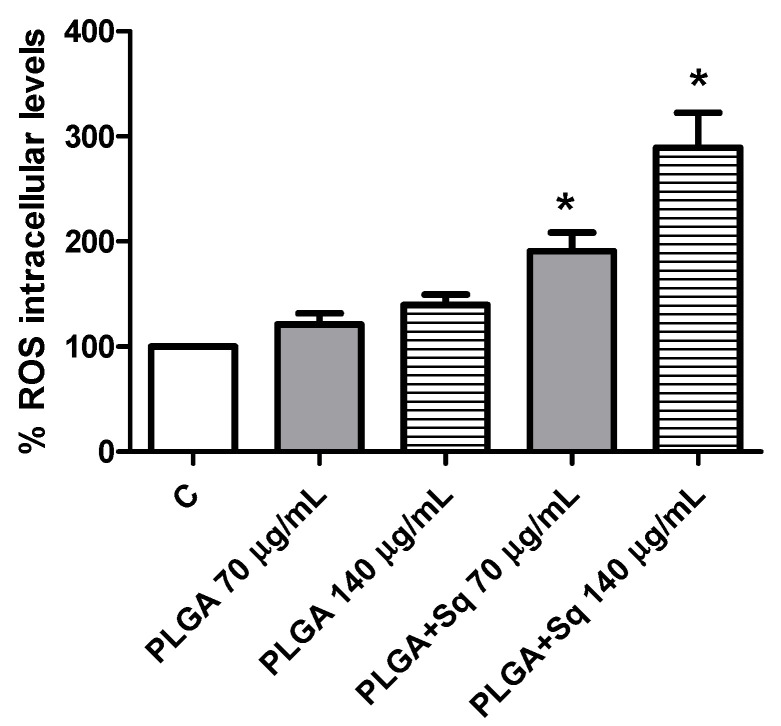
Measurement of ROS levels on undifferentiated Caco-2 cells after 24 h incubation with PLGA + Sq and PLGA alone. PLGA 70 or 140 μg/mL (without Sq) represents the PLGA-NPs required for the indicated Sq concentration. * *p* < 0.05 vs. negative control (without PLGA and Sq). C, control, refers to untreated cells.

**Figure 10:**
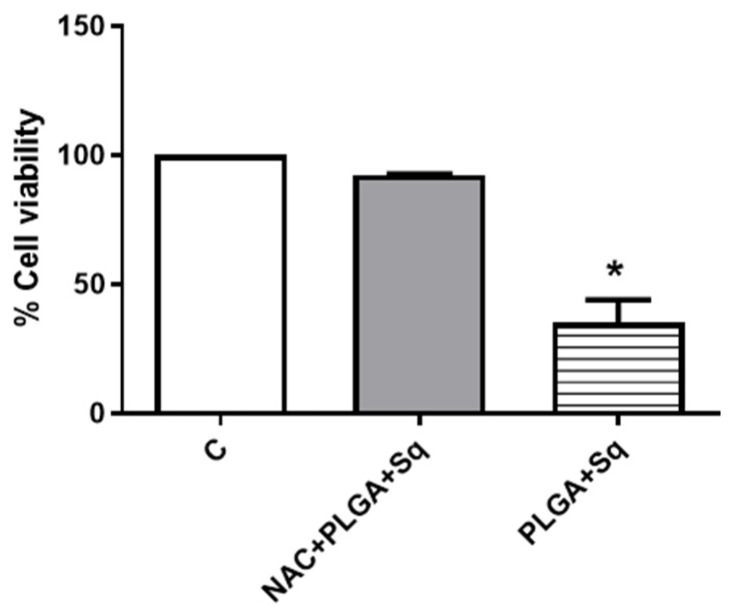
Cell viability measurement after pretreatment of cells with 5 mM NAC for 2 h followed by treatment of cells with 140 μg/mM PLGA + Sq for 72 h. * *p* < 0.05 vs. control. C, control, refers to untreated cells.

Given the possibility that the release of cytochrome c to the cytoplasm may be related to changes in the mitochondrial potential, attributable to alterations in the redox balance, we undertook the measurement of the intracellular levels of ROS. The results demonstrated a significant elevation in ROS levels (Figure 9). It can thus be postulated that the dose-dependent pro-oxidant effect observed of squalene may be the cause of the decrease in cell viability. To substantiate this finding, the cells were incubated with a ROS inhibitor, such as N-acetylcysteine (NAC), at a concentration of 5 mM for 2 h [37]. The results demonstrated that the ROS inhibitor (NAC) effectively reversed the decline in cell viability induced by PLGA + Sq, suggesting that the observed effect may be mediated by the elevation of ROS. (Figure 9 and Figure 10).

**Figure 11:**
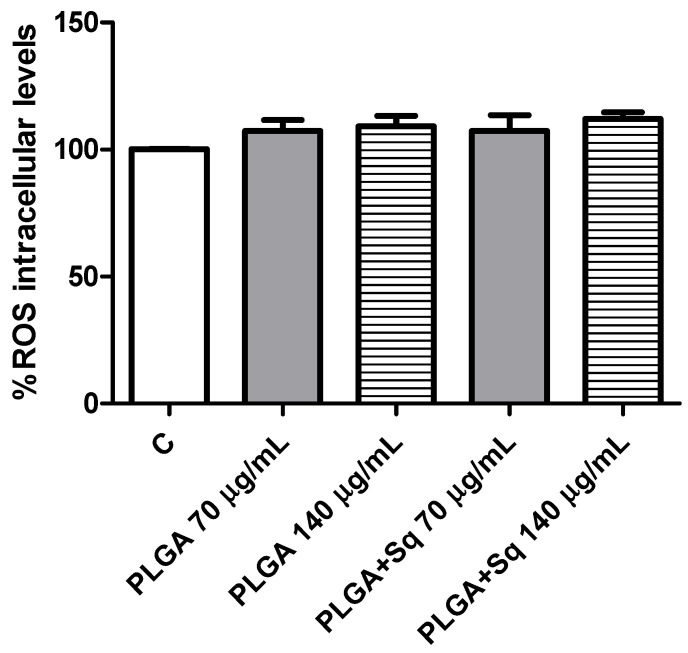
Differentiated Caco-2 cells incubated with 70 or 140 mg/mL PLGA in the presence or absence of squalene. PLGA 70 or 140 μg/mL (without Sq) represents the PLGA-NPs required for the indicated Sq concentration. C, control, refers to untreated cells.

Furthermore, it is crucial to highlight that the presence of squalene in non-cancerous (differentiated) cells did not result in any observable alteration in ROS levels (Figure 11).

## 3. Discussion

The discovery of novel molecular determinants that regulate tumor growth and the development of pharmacological agents that target these molecular pathways may markedly enhance the prognosis and quality of life for different cancer patients [21]. The Mediterranean dietary pattern is now supported by scientific evidence as a means of preventing and managing several diseases. The primary source of fat consumed as part of this dietary pattern is virgin olive oil, which, when regularly consumed, has been shown to reduce the incidence of certain illnesses, including cardiovascular disease and a wide variety of cancers [23,38]. The minor compounds present in this oil may be instrumental in the oil’s salutary properties. Squalene, a crucial minor compound present in virgin olive oil and olives, has been demonstrated to possess antitumor properties against a multitude of cancers, including skin, breast, liver, renal, and colon [22,39,40,41,42]. This article demonstrates that squalene exerts antitumor activity on Caco-2 cells. It is noteworthy that this compound exhibited inhibitory effects on the growth and apoptosis of colon carcinoma cells at specific concentrations, accompanied by an altered cell cycle.

**Figure 12:**
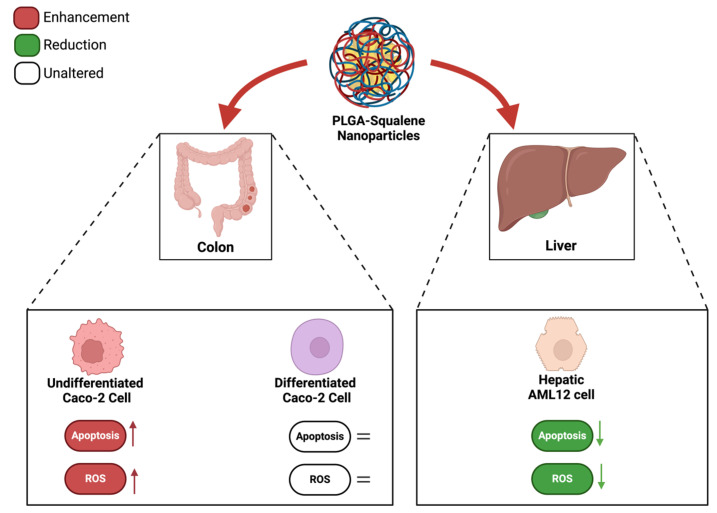
Evaluation of the effect of PLGA + Sq nanoparticles on cytotoxicity and ROS modulation in undifferentiated and differentiated Caco-2 cells, and AML12 cells [16,49].

A number of studies have demonstrated that a variety of anticancer agents, including adriamycin, 5-fluorouracil, hydroxytyrosol, and bleomycin, exhibit enhanced antitumor activity when combined with squalene [39,43,44]. The results of these studies indicate that the combination of squalene and antitumor agents may exert a synergistic effect through two main mechanisms: firstly, by interfering with the efflux of anticancer drugs from cells, and secondly, by modifying the permeability of cell membranes. Indeed, the combination of anticancer agents with membrane-active molecules, such as squalene, could reduce the required dosage of anticancer drugs [39,45,46]. The initial investigation sought to ascertain the potential impact of squalene on the viability of Caco-2 cells. To substantiate the impact of squalene, it was incorporated into PLGA nanoparticles and their capacity to facilitate the transportation of squalene to Caco-2 cells was investigated. As evidenced by multiple studies, nanoparticles made from PLGA, a naturally derived and biodegradable polymer, serve as an effective nanocarrier for the encapsulation and delivery of a wide range of anti-cancer agents, including oleanolic and ursolic acids, haloperidol, and estradiol, in three distinct cell lines: HepG2, Caco-2, and Y-79 [16,47,48]. Dose-response curves were constructed using varying concentrations of squalene in a 72 h preincubation period, delivered with DMEM, DMSO, EtOH, and PLGA. The findings indicated a dose-dependent antiproliferative effect of squalene (exclusive of DMEM), with PLGA-bound squalene demonstrating the most notable reduction in cell viability (Figure 1). Furthermore, these findings indicated that stable squalene NPs were effectively internalized by Caco-2 cells within 24 h (Figure 4). However, the viability dose-response curves over time demonstrated that the effect was more pronounced at longer times (72 h) and higher concentrations (250 μg/mL) (Figure 2). To exclude the potential for PLGA and squalene to exert adverse effects on non-cancerous cells, their viability was evaluated in the presence of the carrier with and without squalene. The results demonstrated that PLGA and squalene did not significantly affect the viability of differentiated cells (Figure 3). Our previous study demonstrated the efficacy of PLGA + Sq nanoparticles in delivering squalene to hepatocytes, establishing these nanoparticles as a superior delivery system compared to other carriers such as chitosan and ethanol. The superior cellular uptake of PLGA + Sq nanoparticles, observed at both 48 h and 72 h, highlights their efficiency in enhancing squalene bioavailability [49]. In addition, PLGA + Sq nanoparticles enhanced cell viability and reduced ROS levels in non-cancerous AML12 cells expressing p53 at the mRNA level (RNA-seq accession number GSE242049), indicating their biocompatibility and potential for therapeutic use [16]. In contrast, Caco-2 cells, which are known to express low levels of p53 [50], showed reduced sensitivity to the lower dose of squalene treatment (Figure 1). This observation lends support to the classic argument that intestinal epithelial cells, such as Caco-2, possess an intrinsic capacity for greater resilience to external stressors. This resilience can be attributed to their natural adaptation to survive significant fluctuations in metabolic activity, pH balance, and osmolarity [3,25,37,51]. Importantly, to ensure the safety of PLGA + Sq nanoparticles, a comprehensive evaluation was performed through an in vitro study, where cytotoxicity and ROS assays in differentiated and undifferentiated Caco-2 cells confirmed that PLGA nanoparticles alone did not induce any adverse effects, further supporting their safety profile (Figure 3, Figure 5, Figure 6, Figure 9 and Figure 11). Taken together, these results provide a basis for the safe and effective use of PLGA + Sq nanoparticles (Figure 12) [16,49]. Consequently, the utilization of PLGA for drug delivery or tissue engineering applications is associated with a minimal risk of systemic toxicity [52,53].

Indeed, ROS, which encompass free radicals such as superoxide anion (O_2_^−^), lipid radicals (ROO), hydroxyl radical (•HO), and non-radicals including hydrogen peroxide (H_2_O_2_), hypochlorous acid (HClO), and peroxynitrite (ONOO^−^), exert a pivotal influence on tumor progression [54,55]. In this context, squalene has been demonstrated to possess potent antioxidant properties within intracellular environments [20,56]. A number of studies have demonstrated that squalene is capable of reducing basal and H_2_O_2_-induced ROS levels in human breast cancer cells and murine macrophages, as well as pulmonary and hepatic cells [16,39,57]. Despite the potential protective effect of squalene against tumor development, the precise mechanism of its selective antioxidant sensitivity remains to be elucidated [39]. Experimental evidence from animal models indicates that squalene may possess tumor-inhibiting properties [58]. It is well established that cancer cells are capable of adapting their metabolism to new stress conditions [10,59]. Nevertheless, once adaptation occurs, alterations in ROS levels (either increment or decrement) might result in the inhibition of cancer growth due to the lack of oxidative stress enzymes [60]. Consequently, under conditions of heightened stress in cancer, squalene is able to induce elevated ROS levels, which could potentially promote the inhibition of colon cancer growth (Figure 9). It may therefore be concluded that the dose-dependent pro-oxidant effect of squalene is responsible for the reduction in cell viability observed. To substantiate this outcome, the cells were treated with a ROS inhibitor, and the findings indicated that in the absence of ROS, squalene was unable to alter the viability of Caco-2 cells (Figure 10). Furthermore, it is crucial to highlight that the presence of squalene in non-cancerous (differentiated) cells does not result in any observable alteration in the levels of ROS (Figure 11). These findings suggest that squalene may exert an inhibitory effect on colon cancer cell growth via ROS pathways.

Furthermore, pathophysiological conditions can precipitate an imbalance between ROS (oxidants) and antioxidant mechanisms within biological systems, resulting in functional disruption [55]. The direct oxidizing effects of ROS on macromolecules, including DNA, proteins, and lipids, can contribute to cell damage, necrotic cell death, and apoptosis [61]. It appears evident that squalene possesses the potential to augment the pro-apoptotic and antiproliferative functions in human colon cancer cells. With regard to this issue, the results demonstrated that squalene induced programmed cell death through both early and late apoptosis, thereby precluding the possibility of death by necrosis, which could give rise to adverse effects such as inflammation (Figure 5). Furthermore, PLGA devoid of squalene did not elicit any discernible effect on the cells. The antioxidant properties of squalene are more pronounced than its anti-inflammatory properties [54]. The protective role of squalene is elucidated by its capacity to regulate cytochrome c expression. Cytochrome c, present in the cytoplasm, initiates the activation cascade of caspases, which ultimately leads to caspase-3, the executioner of apoptosis [62,63]. Figure 6 illustrated that in cells treated with PLGA + Sq, the cytochrome value was lower in the mitochondria, indicating that it had migrated to the cytoplasm. This phenomenon may be associated with apoptosis, as previously observed under these conditions (Figure 5). Similarly, we sought to corroborate this finding by determining the activity of caspase-3, and the results demonstrated a significant increase in caspase-3 activity due to the action of squalene (Figure 7). However, another study indicated that the administration of squalene did not effectively prevent simvastatin-induced caspase-dependent apoptosis in rat neonatal cardiac fibroblasts and myofibroblasts [64]. Moreover, the investigation revealed that squalene did not demonstrate notable scavenging activity and exhibited minimal impact on cell proliferation rates, cell cycle profiles, and cell apoptosis in human breast cancer cells [65].

In addition to squalene, tocotrienol demonstrates the strongest inhibitory effect on cellular proliferation and is linked to the activation of caspase-3, -6, and -7, in addition to causing G1, G2/M, and sub-G1 cell cycle arrest and inter-nucleosomal DNA fragmentation, which are associated with apoptosis in leukemia cells, by depleting intracellular squalene [66]. In order to find the impact of squalene on Caco-2 cells, an investigation was conducted into the expression of genes associated with cell death, inflammation, and the cell cycle (*NLRP3*, *PYCARD*, *BCAR1*, *IL-1B*, *GPX4*, *FAF1*, *RIPK1*, *MLKL*, *ATM*, *CHEK2*, *BECN1*, and *CCND1*). The results demonstrated no alterations in the expression of any of the genes examined, suggesting that the antiproliferative effect of squalene may be post-transcriptional and not directly acting on the mRNA (Table S1, Figure S1). Accordingly, the potential impact of squalene on the cell cycle was examined. The analysis of the cell cycle revealed a modest arrest in the G1 phase, accompanied by a reduction in the S phase, in the PLGA + Sq condition (Figure 8). The precise mechanism by which squalene exerts its effects on cancer cells remains unclear. However, it is postulated that this may involve the translocation of cyclin D1, a protein essential for the G1/S cell cycle transition, to the cytoplasm, where it is degraded [39]. Alternatively, it may result in a reduction in HMG-CoA reductase levels, leading to the induction of apoptosis and cell cycle arrest [67]. In light of the intricate role of p53 in cell cycle regulation and apoptosis [68,69], future studies comparing the effects of squalene encapsulation in PLGA nanoparticles on cancerous cell lines with functional p53 transcription factors could provide further insights into the underlying mechanisms. Such a comparison would assist in determining whether the antiproliferative and pro-apoptotic effects of squalene observed in this study are influenced by p53-mediated pathways.

Nevertheless, PLGA + Sq showed promising potential for colorectal cancer prevention by enhancing the bioavailability and effectiveness of squalene, a compound with antioxidant and anticancer properties. The PLGA nanoparticles improved squalene’s solubility, facilitated targeted delivery to cancerous cells, and minimized systemic toxicity [70,71]. This formulation offers a non-invasive method, making it a viable adjunct to current prevention strategies, particularly for at-risk populations, and could enhance patient compliance in clinical settings [70,72].

## 4. Materials and Methods

### 4.1. The Formulation of Squalene Nanoparticles (PLGA)

As previously described, following the physicochemical characterization of PLGA nanoparticles and the determination of their encapsulation effectiveness, a single-emulsion solvent evaporation method was employed to synthesize the squalene-PLGA polymeric nanoparticles [16]. In summary, 50 mg of Resomer^®^ RG 503H poly (D, L-lactide-co-glycolic) (PLGA-COOH, Mw 24–38 kDa) (Sigma-Aldrich, Merck Millipore, Darmstadt, Germany) and 150 mg of Pluronic F68 (Panreac Química S.L.U, Barcelona, Spain), used as a stabilizing agent to enhance nanoparticle dispersion and stability, were dissolved in 5 mL of ethyl acetate 99.6% ACS (Sigma-Aldrich, Merck Millipore, Darmstadt, Germany). A total of 50 µL of squalene (2.05 M, ≥98%, liquid) (Sigma-Aldrich, Merck Millipore, Darmstadt, Germany) was added to the solution together with 10 mL of Milli-Q water. This solution was then sonicated (Branson Digital Sonifier 450, Danbury, CT, USA) in an ice bath for 25 s at 40% amplitude using a probe of 0.13 inches in diameter. Subsequently, the organic solvent was allowed to evaporate for a period of three hours, during which the solution was stirred at 600 rpm in accordance with sterile conditions. The nanoparticles were dispersed in fresh PBS for the subsequent cellular studies, following their collection by centrifugation (Thermo Fisher Scientific, Waltham, MA, USA) at 12,350 rpm for 15 min at 10 °C, followed by a second centrifugation at 15,000 rpm for 15 min at 10 °C. The synthesis of PLGA + Sq nanoparticles with an estimated diameter of approximately 170 nm demonstrated an encapsulation efficiency of 77%, as previously reported [16].

### 4.2. Cell Culture

The human cell line Caco-2 (clone TC7) was kindly provided by Dr. Edith Brot-Laroche (Université Pierre et Marie Curie-Paris 6, UMR S 872, Les Cordeliers, Paris, France). Cells were maintained in a humidified atmosphere of 5% CO_2_ at 37 °C. They were cultured in Dulbecco’s modified Eagle’s medium (DMEM) (Gibco Invitrogen, Paisley, UK) supplemented with 20% fetal bovine serum (FBS), 1% non-essential amino acids, 1% penicillin (1000 U/mL), 1% streptomycin (1000 μg/mL), and 1% amphotericin (250 U/mL). Cells were enzymatically passaged with 0.25% trypsin-1 mM EDTA and sub-cultured into 25 cm^2^ plastic flasks at a density of 5 × 10^5^ cells/cm^2^. The culture medium was replaced every 2 days. The treatment with squalene by using different carriers was carried out 24 h after sowing undifferentiated cells. The percentage of cell confluence was determined by observation with light microscopy and when they reached 80% confluence, the cells differentiated into normal enterocytes.

### 4.3. Squalene Extraction from Caco-2 Cells

The cell sample was weighed and then homogenized with 1 mL of PBS and 10 µL of the standard 5α-cholestane (1.75 mM) (Sigma-Aldrich, Merck Millipore, Darmstadt, Germany). The subsequent step involved the addition of 2 mL of cyclohexane (Honeywell, Muskegon, MI, USA). The mixture was thoroughly combined and then subjected to centrifugation at 4000 rpm for 10 min. The upper phase was then transferred to a new Falcon tube. A volume of 1.5 mL of cyclohexane (Honeywell, Muskegon, MI, USA) was added to the initial Falcon container, mixed thoroughly, and centrifuged at 4000 rpm for 10 min. Subsequently, the upper phase was collected and combined with the preceding one. A tablespoon of silica (Macherey-Nagel, Duren, Germany) was added to the Falcon container where the upper phases had been collected and then the tube was vortexed. The mixture was then subjected to centrifugation at 4000 rpm for 10 min, after which 2 mL of the upper phase were collected and transferred to a glass tube. The samples were thereafter dried in a nitrogen atmosphere. The solution was then reconstituted with 200 µL of squalene (Sigma-Aldrich, Merck Millipore, Darmstadt, Germany) at a concentration of 50 µM, and the mixture was subjected to three minutes of ultrasound water bath. The concentration of squalene was subsequently analyzed by gas chromatography and mass spectrometry without any saponification procedures [16,17]. In brief, chromatographic analyses were performed using an Agilent 6890 CG system, comprising a 7683B injector and a 5975B MS acquisition parameter unit (Agilent Technologies, Santa Clara, CA, USA). A J&W 122-5532 column (Agilent Technologies, Santa Clara, CA, USA) with a nominal length of 30 m and a diameter of 0.25 mm was employed, with a flow of helium at 1 mL/minute. The oven temperature was set to run from 280 to 290 °C in 15 min with a ramp from 5 to 13 min.

### 4.4. Cell Death Studies

For cytotoxicity assays, Caco-2 cells were seeded in 96-well plates at a density of 4 × 10^3^ cells/well. The culture medium was replaced with different vehicles (DMEM, DMSO, EtOH, and PLGA with squalene) 24 h after seeding and the cells were incubated for 72 h. An initial range of varied concentrations from 50 to 250 µg/mL was used and the IC_50_ value was determined for the vehicle showing the greatest decrease in cell viability. The PLGA vehicle was selected, and dose-response curves were performed at different times (24, 48, and 72 h). The antiproliferative effect was measured with the MTT assay as previously described by Quero et al. [73]. The absorbance at 540/620 nm was measured with the SPECTROstar Nano (BMG Labtech, Ortenberg, Germany).

### 4.5. Apoptosis Determination by Flow Cytometry

For the apoptosis assays, cells were seeded in 25 cm^2^ flasks and exposed to PLGA + Sq at the IC_50_ concentration for 72 h. Cells were then harvested, stained with annexin V-FITC and propidium iodide, and analyzed as previously described [51]. Negative and positive controls were included, with untreated cells serving as the negative control and PLGA-treated cells as the positive control. Briefly, after incubation, cells were transferred to flow cytometry tubes and washed twice with phosphate buffered saline (PBS), followed by resuspension in 100 µL of annexing V binding buffer (100 mM Hepes/NaOH (pH 7.4), 140 mM NaCl, 2.5 mM CaCl_2_). A total of 5 µL of annexin V-FITC and 5 µL of propidium iodide were added to each tube. After a 15 min incubation at room temperature in the dark, 400 µL annexin-binding buffer was added and analyzed by flow cytometry (Beckman Coulter, Brea, CA, USA) for 1 h. Signal intensity was measured with a BD FACSAria^TM^ Cell Sorter (BD Biosciences, San Jose, CA, USA) and analyzed with BD FACSDiva^TM^ version 8.0 Software (BD Biosciences, San Jose, CA, USA).

### 4.6. The Determination of Cytochrome C and Caspase-3 by Flow Cytometry

Caco-2 cells were seeded in 25 cm^2^ flasks at a density of 3 × 10^5^ cells per flask and incubated for 24 h under standard cell culture conditions. Then, cells were incubated with the IC_50_ (140 μg/mL) of PLGA+ Sq for 72 h. Cells with released cytochrome c were analyzed according to Christensen et al. [74] with a slight modification [51]. The caspase-3 activity was analyzed following the protocols shown by Quero et al. [75].

### 4.7. Propidium Iodide Staining of DNA Content and Cell Cycle Analysis

Once the cells were seeded in 25 cm^2^ flasks at a density of 1 × 10^4^ cells/cm^2^, they were exposed, 24 h post-shipment, to the study panel (control, PLGA, and PLGA + Sq) for 72 h at the IC_50_ concentration (140 μg/mL). Cells were fixed and analyzed according to previously described methods [50].

### 4.8. RNA Extraction and Quantitative Real-Time PCR

A Quick-RNA^TM^ MiniPrep kit was used to extract the total cellular RNA in accordance with the manufacturer’s recommendations (Zymo Research, Irvine, CA, USA). Subsequently, RNA quality was evaluated based on the absorbance ratio at 260/280 nm wavelength using a Nanodrop 2000c Spectrophotometer (Thermo Fisher Scientific, Waltham, MA, USA). The integrity of the 28S and 18S ribosomal RNAs was validated by electrophoresis on a 1% agarose gel, followed by ethidium bromide staining, and the 28S/18S ratio was found to be greater than 2.

The reverse transcriptase quantitative PCR testing of these transcripts was optimized for the primers and input cDNA quantities in order to achieve equal efficiency. In accordance with the manufacturer’s instructions, 500 ng of the extracted total RNA was reverse transcribed into supplemental deoxyribonucleic acid using the PrimeScript^RT^ reagent kit (TaKaRa Biotechnology, Kusatsu, Shiga, Japan) in the presence of random and oligo (dT) primers. The primers for each gene were designed using Primer Blast (NCBI) (Bethesda, MD, USA), as detailed in Supplementary Table S1. Following this, the primers were verified for gene specificity and the amplification of cDNA, rather than genomic DNA, using BLAST analysis. Quantitative real-time PCR was conducted on a Step One Plus Real-Time PCR System (Applied Biosystems, Foster City, CA, USA) in accordance with the manufacturer’s instructions (SYBR Green PCR Master Mix, Applied Biosystems, Foster City, CA, USA). The relative ratio of each gene’s transcript expression level to the mean values of control samples was calculated, using the comparative 2^−ΔΔCT^ method with normalization to the *TBP* endogenous control gene, and expressed for each gene.

### 4.9. The Determination of Intracellular Levels of ROS

Cells were seeded in 96-well plates at a density of 4 × 10^3^ cells/well. The intracellular level of ROS was assessed using the dichlorofluorescein assay, as previously described [51]. Cells were cultured for 24 h before being incubated with/without PLGA or PLGA + Sq for 24 h. Subsequently, the medium was removed, and the cells were washed twice with PBS and incubated for 1 h with 20 µM of 2′,7′-dichlorofluorescein diacetate (DCFH-DA) in PBS at 37 °C. The formation of the fluorescence-oxidized derivative of DCF was monitored at an emission wavelength of 535 nm using an excitation of 485 nm on a FLUOstar Omega multiplate reader (BMG Labtech, Ortenberg, Germany). ROS levels were measured by assessing the fluorescence at time “zero” and after 20 min of incubation at 37 °C. Fluorescence intensity values were expressed as a percentage compared to the control, reflecting the total ROS content. The same procedure was applied to the differentiated Caco-2 cells after they reached confluence.

### 4.10. Statistical Analysis

All tests were performed at least three times. Data are presented as mean ± SD using a one-way analysis of variance (ANOVA). Significant differences at *p* < 0.05 were compared using the Bonferroni multiple comparisons test and Mann–Whitney U test. Statistical analysis and graphs were performed using GraphPad Prism version 5.02 on a PC (La Jolla, CA, USA).

## 5. Conclusions

The present study highlights the potential of PLGA-encapsulated squalene as a novel approach for inhibiting the proliferation of colon carcinoma cells, particularly in Caco-2 cells. These results demonstrated a dose-dependent antiproliferative effect, with the PLGA formulation exhibiting the most pronounced inhibition compared to other carriers such as DMEM, DMSO, and EtOH. Notably, squalene had a minimal impact on the viability of differentiated cells, suggesting its specificity for cancerous cells. Importantly, squalene nanoparticles were successfully internalized by Caco-2 cells within 24 h, enhancing the efficacy over time at the highest concentrations tested. Mechanistically, squalene exerted its inhibitory effects through the generation of ROS and induction of apoptosis via cytochrome c release and caspase-3 activation. The cell cycle analysis further suggested that squalene treatment led to a modest accumulation of cells in the G1 phase, with a corresponding decrease in the S phase. The results of the impact of squalene on genes associated with cell death, inflammation, and the cell cycle indicate that the antiproliferative effect of squalene may be post-transcriptional and not directly acting on RNA. Overall, these findings demonstrate the promising potential of PLGA-encapsulated squalene as an effective, targeted strategy to enhance the antiproliferative effects of squalene on colon cancer cells through apoptosis and redox imbalance disruption. This novel formulation could be explored as a preventive therapeutic approach for colorectal cancer, offering a potential alternative for enhancing cancer treatment efficacy while minimizing toxicity.

## Data Availability

Data are contained within the article and the Supplementary Materials.

## Supplementary Materials

The following supporting information can be downloaded at: https://www.mdpi.com/article/10.3390/ijms252313048/s1.

## Abbreviations

DMEM	Dulbecco’s modified Eagle’s medium
DMSO	Dimethyl sulfoxide
DNA	Deoxyribonucleic acid
ddH_2_O	Deionized distilled H_2_O
DCFH-DA	2′,7′-dichlorofluorescein diacetate
EtOH	Ethanol
EDTA	Ethylenediaminetetraacetic acid
FBS	Fetal bovine serum
GC/MS	Gas chromatography/mass spectrometry
HMG CoA	3-hydroxy-3-methylglutaryl coenzyme A
mRNA	Messenger RNA
NPs	Nanoparticles
NAC	N-acetylcysteine
PLGA	Poly (lactic-co-glycolic acid)
PBS	Phosphate-buffered saline
ROS	Reactive oxygen species
RT-qPCR	Quantitative reverse transcription -PCR
RNA	Ribonucleic acid
Sq	Squalene
