# Supplementary material for: Squalene in Nanoparticles Improves Antiproliferative Effect on Human Colon Carcinoma Cells Through Apoptosis by Disturbances in Redox Balance
Source: Int J Mol Sci. 2024 Dec 4;25(23):13048. doi: 10.3390/ijms252313048 (PMC11641743; doi:10.3390/ijms252313048)
Supplement: Supplementary file 1 [file ijms-25-13048-s001.zip › ijms-3333463-supplementary.pdf]

Article

Squalene in Nanoparticles Improves Antiproliferative Effect on Human Colon Carcinoma Cells Through Apoptosis by Disturbances in Redox Balance

Seyed Hesamoddin Bidooki [1], Javier Quero [2], Javier Sánchez-Marco [1], Tania Herrero-Continente [1], Inés Marmol [2], Roberto Lasheras [3], Victor Sebastian [4,5,6], Manuel Arruebo [4,5], Jesús Osada [1,7,8] and María Jesús Rodriguez-Yoldi [2,7,8,*]

1 Departamento de Bioquímica y Biología Molecular y Celular, Facultad de Veterinaria, Instituto de
Investigación Sanitaria de Aragón, Universidad de Zaragoza, E-50013 Zaragoza, Spain; h.bidooki94@gmail.com (S.H.B.); javiersanchezmarc@gmail.com (J.S.-M.); taniaherrero1992@gmail.com (T.H.-C.); josada@unizar.es (J.O.)

2 Departamento de Farmacología, Fisiología, Medicina Legal y Forense, Facultad de Veterinaria, Instituto de Investigación Sanitaria de Aragón, Universidad de Zaragoza, E-50013 Zaragoza, Spain;
javierquero94@gmail.com (J.Q.); ines.marmol@gmail.com (I.M.)

3 Laboratorio Agroambiental, Servicio de Seguridad Agroalimentaria de la Dirección General de
Alimentación y Fomento Agroalimentario, Gobierno de Aragón, E-50071 Zaragoza, Spain; rjlasheras@aragon.es

4 Departamento de Ingeniería Química y Tecnologías del Medio Ambiente, Universidad de Zaragoza,
E-50018 Zaragoza, Spain; victorse@unizar.es (V.S.); arruebom@unizar.es (M.A.)

5 Instituto de Nanociencia y Materiales de Aragón (INMA), CSIC, Universidad de Zaragoza, E-50009 Zaragoza, Spain

6 Centro de Investigación Biomédica en Red de Bioingeniería, Biomateriales y Nanomedicina (CIBER-BBN), Instituto de Salud Carlos III, E-28029 Madrid, Spain

7 Instituto Agroalimentario de Aragón, CITA, Universidad de Zaragoza, E-50013 Zaragoza, Spain

8 CIBER de Fisiopatología de la Obesidad y Nutrición, Instituto de Salud Carlos III, E-28029 Madrid, Spain

* Correspondence: mjrodyol@unizar.es; Tel.: +34-976-761649

Table S1. Sequences of real-time PCR primers according to MIQE guidelines

Gene symbol	Primer sequence, sense/antisense ((5'-3')	Amplicon length	Accession	Exon
ATM	AGCGCCTGATTCGAGATCCT<br>CCTGGAGGCTTGTGTTGAGG	179	XM_047426981.1 , XM_011542843.3 ,XM_047426979.1 , XM_047426978.1 , XM_047426977.1 , XM_011542842.4 , XM_047426976.1, NM_001351834.2 XM_017017790.3, XM_011542840.4 , XM_005271562.6 , XM_047426975.1 , XM_006718843.5 , NM_001351836.2 , NM_001351835.2 , NM_000051.4	3/4
CHEK2	GGACTGCTGGGTATAACCGTG<br>AGGTGGATACCCACTAAGGCA	80	XM_011529845.3, NM_145862.2, XM_047441106.1, XM_047441104.1, XM_047441105.1, NM_001349956.2, NM_007194.4, NM_001257387.2, NM_001005735.2, XM_006724116.3 XM_011529839.3, XM_024452149.2, XM_024452148.2, XM_047441107.1, XM_011529840.4, XM_011529842.3, XM_017028560.2, XM_047441108.1, XM_006724114.4,	12/13
BECN1	GGAAGGGTCTAAGACGTCCA<br>CTCGTGTCCAGTTTCAGGGG	87	XM_005257759.4, XM_005257760.5, XM_017025263.3, XM_017025264.3, NM_001313999.1, NM_001314000.2, NM_001313998.2, NM_003766.5	2
CCND1	AGCTGTGCATCTACACCGAC<br>GAAATCGTGCGGGGTCATTG	113	NM_053056.3	2/3
NLRP3	GAGAGAGCTGCGATCCATCC<br>CCCGATGCTGTCATTGTCCT	158	XM_047443535.1, XM_024452862.2, XM_024452874.2, XM_047443539.1, XM_047443533.1, XM_047443557.1, XM_047443546.1, XM_047443571.1, NM_001243133.2, NM_004895.5, NM_001127461.3 XM_047443578.1, NM_001127462.3, NM_183395.3, XM_047443582.1, XM_017000181.2, XM_017000182.2, XM_047443562.1, NM_001079821.3, XM_047443534.1	9
PYCARD	ACAAACGTTGAGTGGCTGCT<br>TTCCGCATCTTGCTTGGGTT	104	NM_145182.3, NM_013258.5	2
BCAR1	GCATCTGAGTTGGGAGGGAG<br>GATCAGGAGGGCTTGCGAAT	231	NM_001170715.3, NM_001170716.3, NM_001170718.3, NM_001170720.3, NM_001170714.3, NM_014567.5, NM_001170717.3, NM_001170719.3, NM_001170721.3	6
IL-1B	GGATCTCCTGTCCATCAGCC<br>GTCAGGCGGGCTTTAAGTGA	115	NM_000576.3, XM_047444175.1	7
GPX4	CAGTGAGGCAAGACCGAAGT<br>CCGAACTGGTTACACGGGAA	104	NM_001039847.3, NM_002085.5, NM_001367832.1, NM_001039848.4	3
FAF1	CCAGGCCCAGTAAGTGAGTC<br>GCACATCCAGCAGTCCAAGA	135	NM_007051.3, XM_047442745.1, XM_047442743.1, XM_024452734.2, XM_024452736.2	19
RIPK1	AATGGCGGCACCCTCTACTA<br>ACTTCTCTGTGGGCTTTGCG	70	XM_047419447.1, XM_047419448.1, NM_001317061.3, NM_001354932.2, NM_003804.6, NM_001354931.2, XM_017011405.2, NM_001354934.2, NM_001354933.2, NM_001354930.2, XM_006715237.4 XM_047419445.1, XM_047419446.1, XM_017011403.2, XM_017011404.3	4
MLKL	ACTGTGCACGGGACAGATTG<br>ACACCGTTTGTGGATGACCT	103	XM_005255834.5, NM_001142497.3, NM_152649.4, XM_047433704.1, XM_047433710.1, XM_047433707.1, XM_047433705.1, XM_047433708.1, XM_047433709.1, XM_047433706.1, XM_011522936.3	1/2
TBP	TGCTGCGGTAATCATGAGGA<br>TGGACTGTTCTTCACTCTTGGC	101	NM_001172085.2, NM_003194.5	4/5

Abbreviations: *ATM,* ATM serine/threonine kinase; *CHEK2,* checkpoint kinase 2; *BECN1,* beclin 1; *CCND1,* Cyclin D1; *NLRP3*, NLR family pyrin domain containing 3; *PYCARD,* PYD and CARD domain containing; *BCAR1,* BCAR1 scaffold protein, Cas family member; *IL-1B*, interleukin 1 beta; *GPX4,* glutathione peroxidase 4; *FAF1,* Fas associated factor 1; *RIPK1,* receptor interacting serine/threonine kinase 1; *MLKL,* mixed lineage kinase domain like pseudokinase; TBP, TATA-box binding protein

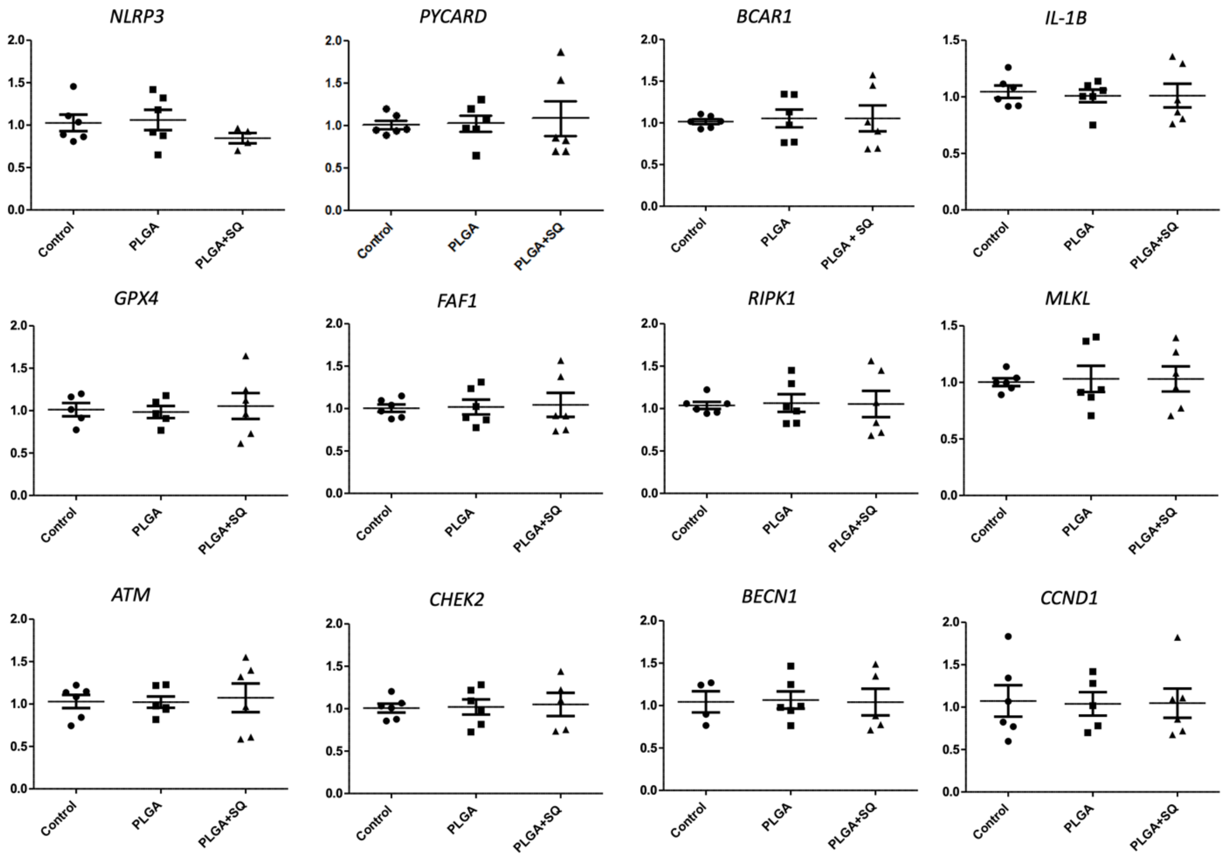

Figure S1. The effect of PLGA+Sq at the IC_{50} concentration [140 μg/mL] for an incubation period of 72 hours on the mRNA expression of genes associated with cell death, inflammation, and the cell cycle in Caco-2 cells. Y-axis refers to the “Relative fold change”.
